# The Microbiome of Catfish (*Ictalurus punctatus*) Treated with Natural Preservatives During Refrigerated Storage

**DOI:** 10.3390/microorganisms13020244

**Published:** 2025-01-23

**Authors:** Jung-Lim Lee, Gregory Yourek

**Affiliations:** 1 Food Science and Biotechnology Program, Food Microbiology Laboratory, College of Agriculture Science and Technology, Delaware State University, Dover, DE 19901, USA; 2 Delaware Nucleotide Analysis (DNA) Core Center, Delaware State University, Dover, DE 19901, USA

**Keywords:** catfish, 16S metagenomics, microbial spoilage, food quality, vinegar, lemon, grapefruit

## Abstract

Fish is an essential lean protein source worldwide. Unfortunately, fresh fish food products deteriorate rapidly due to microbial spoilage. With consumers’ growing concerns about using chemical preservatives, we propose using natural preservatives as safer alternatives to prevent microbial spoilage. In this study, we used Next-Generation Sequencing (NGS) metagenomics to study microbiomes on catfish fillets at early (day one for all samples), middle (day seven for control store-bought and aquaculture-raised samples, day nine for other treatment store-bought samples, and day eleven for other treatment aquaculture-raised samples), and late (day fifteen for all store-bought, day eleven for control aquaculture-raised samples, and day twenty-seven for other treatment aquaculture-raised samples) points. Store-bought and aquaculture-raised catfish were treated individually with natural preservatives (vinegar, lemon, and grapefruit seed [GSE]). We observed bacterial populations and sequenced 16S NGS libraries of catfish microbes. Vinegar treatment showed the greatest suppression of bacterial growth in both groups, and GSE and lemon treatment had similar levels of suppression in the mid and late points (−4 to −5 Log CFU/g vinegar and −0.1 to −4 Log CFU/g other treatments in aquaculture and −1 to −2 Log CFU/g vinegar and −0.2 to −0.5 Log CFU/g other treatments in store-bought). Aquaculture-raised vinegar treatment samples had similar proportional taxonomy abundance values through storage duration. *Pseudomonas*, *Janthinobacterium*, and *Camobacteriaceae* were the dominant bacteria species in the early point for store-bought fish. Still, *Pseudomonas* was suppressed by vinegar treatment in the middle point, which allowed for less biased relative abundance compared to other treatments. *Chryseobacterium*, CK-1C4-19, and *Cetobacterium* were the dominant bacteria species for early point treatments in aquaculture-raised fish. Still, they remained the predominant bacteria for only aquaculture-raised vinegar samples in the middle and late points, which allowed for a similar relative abundance to fresh catfish. Meanwhile, *Pseudomonas* in most lemon and GSE samples became the dominant species at a later point. This study provides a better understanding of bacterial spoilage of catfish during storage. Additionally, we showed that natural preservative treatments can effectively extend the shelf-life of fishery products.

## 1. Introduction

Fish is an important diet component worldwide because of its high protein, vitamin, and mineral content, and consuming fish at least one to two times per week is important for human health [1], increasing the demand for high-quality fresh seafood. The channel catfish (*Ictalurus punctatus*) is North America’s most abundant catfish species that consumers widely accept [1]. This has led to a rapid expansion of aquaculture in the United States to increase accessibility to consumers. Since the shelf life of fresh fish ranges from only seven to eleven days with traditional packaging, new techniques are needed to extend this shelf life [2]. The aquaculture of freshwater fish depends on water sources. Lakes, rivers, streams, reservoirs, and surface run-off can contaminate ponds where fish are commercially raised for human consumption [3]. One of the sources of contamination are pathogenic microbes whose survival is affected by temperature fluctuations and other environmental factors [3]. In this study, we compared the bacterial load of catfish products from two dissimilar sources to determine the differences because of fish husbandry and processing techniques.

Fresh fish products spoil more quickly than other foods because of microbial contamination, water activity, and pH [4]. It is estimated that, worldwide, between 30 and 35 percent of fish is lost or wasted every year, primarily due to spoilage [5]. This increases to almost 50 percent in North America. Many microbes in nature are unculturable using traditional methods [6]. Conventional culturing methods are unreliable during complete microbial characterization and identification from foods [7]. Alternatively, the process of choice for analyzing the composition of bacterial communities is sequencing 16S rRNA [8], which covers hypervariable regions of the gene.

Fish producers commonly use preservatives to combat spoilage bacteria [9]. In recent years, consumers have increased interest in natural preservatives. They want foods that are not only free from Specific Spoilage Organisms (SSOs) and foodborne pathogens but are also less processed and contain fewer added “chemical ingredients” [10,11]. Consumers’ avoidance of foods treated with chemical preservatives they deem “artificial” has stimulated interest in developing alternative approaches [7,8].

Organic monoprotic acids, such as vinegar, lemon, and grapefruit, can be easily applied to fish products through simple incremental addition of either raw use or marinades [12]. We have previously shown the antimicrobial effect of vinegar on catfish [1]. Grapefruit seed extract (GSE) is a natural preservative with antimicrobial properties and has been shown to extend the shelf life of fish products [13]. Additionally, lemon juice inhibits microbe growth in seafood products [14].

The microbial community on a food product changes due to the microbes’ tolerance/intolerance to preservatives [15], which could be tracked through Next-Generation Sequencing (NGS). Next-Generation Sequencing produces high-throughput sequencing data from many samples simultaneously in flow cell platforms [16] with applications in target sequencing, whole-genome sequencing (WGS), and 16S metagenomics study which covers hypervariable regions of the gene [17]. A 16S metagenomics is less read-intensive than WGS as it allows loading more samples per sequencing run making it profound for analyzing the composition of bacterial communities [8] and their diverse structures in various foods, agricultural products, and environments [17].

The purpose of this project is to use NGS to study catfish spoilage and its prevention. Our objectives are to (1) determine which natural preservative is most effective against bacterial growth on fish filets and use NGS, to (2) investigate the dynamics of bacterial communities that grow on store-bought vs. naturally raised fish during storage, and to (3) compare the effectiveness of different natural preservatives on different bacterial communities. By exposing store-bought and aquaculture-raised catfish to different preservative treatments, we provide a better understanding of bacterial spoilage on catfish during storage. In this study, we explored the different microbiomes present on catfish fillets after treatment with three natural preservatives (Supplementary Figure S1). While other research on seafood/fishery products focuses on the gut microbiome, we discuss the portion of the product that consumers would eat. Additionally, this study shows the potential of natural preservative treatment to effectively extend fishery products’ shelf-life.

## 2. Materials and Methods

### 2.1. Minimum Inhibitory Concentration of Natural Preservatives

From our literature reviews, six type strains representing spoilage organisms (*Shewanella algae* ATCC 51192, *Shewanella baltica* NCTC10735, *Aeromonas hydrophila* ATCC 7966, *Aeromonas salmonicida* ATCC 33658, *Pseudomonas aeruginosa* ATCC 10145, and *Pseudomonas fluorescens* ATCC 13525) were chosen and inoculated into 5 mL of Tryptic Soy Broth (TSB) (Carolina Biological Supply Company, Burlington, NC, USA) and incubated at 26 °C for 18 h. A subculture was made in 2 mL of TSB and incubated for 6 h. The optical density was adjusted to a consistent concentration of the bacteria (5 × 10^5^ CFU/mL per well) at 595 nm. Six different natural preservatives (GSE, lemon, vinegar, green tea, ginger, and garlic) in various concentrations (0.5–50 µL/100 µL) were added to each bacteria culture at differing concentrations in a 96-well plate. The samples were incubated at 26 °C for 18 h, and the optical density was read at 595 nm. After evaluating the data based on the lowest concentration with no visible bacterial growth, three natural preservatives were chosen for the remainder of the study based on their ability to inhibit bacterial growth [1].

### 2.2. Bacterial Sampling—Store Samples

Our methods were adapted from our previous study [18]. A “fresh” commercial North American catfish (*Ictalurus punctatus*) fillet was acquired from a local retail source, cut up, and 20 g of fish muscle was placed into individual sterile bags. A total of 64 bags were divided into four groups: one for control and three separate groups for natural preservatives lemon (*v*/*v*) (6.7% acidity) 8% (0.54% final acidity), vinegar (*v*/*v*) (5% acidity) 4% (0.2% final acidity), and 8% GSE (*v*/*v*) (NutriBiotic, Lakeport, CA, USA, 2.6% concentration). All the bags designated for the natural preservatives contained catfish treated with each natural preservative before being stored at 4 °C for 30 min. The natural preservatives were drained, and the remaining amounts were rinsed with sterile 0.85% saline solutions before the experiment. Except for the samples in “Day 1 bags”, including the control, the other bags were stored at 4 °C for the remainder of the storage periods. The samples in the bags were stomached with 80 mL saline for 50 s at medium speed. All the liquid from the fish juice was pipetted out for the downstream sampling procedures. Besides a small portion of the fish juice for CFU enumeration, the remaining fish juice was centrifuged at 1300× *g* for 3 min to collect any fish debris at the bottom. Avoiding any fish tissues, the supernatant was transferred and centrifuged at 18,000× *g* for 5 min to collect a bacteria pellet at the bottom. The supernatant was discarded, and the pellet was washed in sterile 0.85% saline solution twice and then stored at −80 °C until DNA was to be extracted. This process was repeated every other day until the bacterial growth curve reached the stationary phase, which was day fifteen.

### 2.3. Bacterial Sampling—Aquaculture-Raised Samples

Our methods were adapted from our previous study [18]. The aquaculture catfish samples were prepared using the same methods as above. The entire process was repeated with North American catfish from the Delaware State University Aquaculture facility. The catfish fillets were obtained from the aquaculture facility and were transferred on ice to the food microbiology lab. The fillets were aseptically sampled and treated with natural preservatives. A total of 288 bags of catfish fillets (20 g) were stored at refrigerated temperatures. From day one, fillets were taken every other day, and fish juice was prepared to collect bacterial pellets and CFU measurements until the stationary point on Day 27.

Twenty-four bacterial samples representing sample treatment and control group, per fish type, storage of early (day one all samples), middle (day seven for control store-bought and aquaculture-raised samples, day nine for other treatment store-bought samples, and day eleven for other treatment aquaculture-raised samples), and late (day fifteen for all store-bought, day eleven for control aquaculture-raised samples, and day twenty-seven for other treatment aquaculture-raised samples) points from the store-bought and aquaculture-raised catfish were sequenced on the Illumina MiSeq for metagenomics analysis. This section of each point was based on the bacterial growth curve and the sensory properties (Supplementary Figure S4) for each sample.

### 2.4. Growth Curve for Total Bacteria

The stomached fish juice was serially diluted with TSB. Select dilutions were applied to Tryptic Soy Agar (TSA) (Carolina Biological Supply Company, Burlington, NC, USA) plates in triplicate and incubated at 27 °C for 120 h. Bacterial colonies were enumerated, and the Log CFU/g was calculated. Each day was plotted, and a bacteria growth curve was generated for the control and the three natural preservative treatments [18]. This process was repeated every other day until the stationary point was reached on day fifteen from the store-bought and day twenty-seventh from the aquaculture-raised catfish.

### 2.5. DNA Extraction, Quantification, and PCR Confirmation

Bacterial genomic DNA was extracted using a Power Food Microbial DNA Isolation kit (Qiagen, Germantown, MD, USA), followed by quantification with a Qubit fluorimeter 3.0 (Thermo Fisher Scientific, Waltham, MA, USA). We also performed the PCR to confirm that the target hypervariable region had been amplified (~550 bp length) [18].

### 2.6. Library Preparation

We performed library preparation according to the manufacturer’s recommendations [19]. In brief, 12 ng of each DNA sample were thoroughly mixed with Kappa HiFi master-mix (Kappa Biosystems Ltd., Cape Town, South Africa), Illum16S-F (TCGTCGGCAGCGTC AGATGTGTATAAGAGACAGCCTACGGGNGGCWGCAG), Illum16S-R primer (GTCTCGTGGGCTCGGAGATGTGTATAAGAGACAGGACTACHVGGGTATCTAATCC), and PCR water (MoBio, Carlsbad, CA). A formulated PCR reaction was performed according to the Illumina library preparation guide using a T100 thermal cycler (Bio-Rad Laboratories, Hercules, CA, USA). The resulting PCR product was cleaned and confirmed on a Bioanalyzer (Agilent Technologies, Santa Clara, CA, USA). Then, Nextera XT indices (Illumina Inc., San Diego, CA, USA) were tagged to the DNA samples through second-round index PCR following the protocols in the library preparation guide. The index PCR products were cleaned and confirmed using a Bioanalyzer. The products were quantified by the Qubit fluorimeter, normalized by dilution with PCR water, and pooled to a microcentrifuge tube before the samples were denatured using NaOH, and a 5% PhiX spike-in (Illumina Inc., San Diego, CA, USA) was added to ensure quality.

### 2.7. 16S rRNA Sequencing

Samples were loaded onto a V3 kit (Illumina Inc., San Diego, CA, USA) and sequenced for a 3-day runtime using an Illumina MiSeq (Illumina Inc., San Diego, CA, USA) system [19].

### 2.8. Metagenomics Data Analysis

After the sequencing, paired FASTQ files for forward- and reverse-sequence reads per sample were generated in the BaseSpace cloud database. The collected sequencing data in FASTQ format were processed and analyzed with the QIIME2 (ver. 2023.2) [20] next-generation microbiome bioinformatics pipeline for metagenomics study. All raw input data were transformed into QIIME2 artifacts (.qza format), which contain information about the data types and sources for downstream pipelines. Following QIIME 2 artifacts generation, visualizations (.qzv format) were created for a statistical table, static images, and any combination of visual data representations. The raw sequencing reads were converted to .qza format with “Casava 1.8 paired-end demultiplexed fastq”. Then, the amplicon denoising and filtering process with the DADA2 plugin was executed to remove potential base errors and chimeric sequences, construct denoised paired-end sequences, and dereplicate them [21]. As a result, a feature table and rep-seqs were generated.

All the core metrics used for alpha and beta diversity analysis were computed based on the rooted phylogenetic tree. Proportional taxonomy abundance was calculated with the metrics for Shannon, observed features, evenness, and Faith’s phylogenetic diversity vectors [22] at the sample depth of 12,386. Beta diversity was achieved for qualitative (Jaccard and unweighted UniFrac) and quantitative distance matrices (Bray–Curtis and weighted UniFrac) at the sample depth of 12,386 to normalize sample diversity. We used Emperor on QIIME2 view to generate principal coordinates analysis plots (PCoA) to investigate sample distances in a 3D structure. Alpha rarefactions were plotted and created with a rooted tree and table at the sequencing depth 15,000. We trained the Naive Bayes classifier using Greengenes 13_8 reference sequences and classified the representative sequences created with the q2-feature-classifier plugin. Taxonomic levels of bacterial classification were represented in bar graphs generated by the QIIME2 taxa plugin. A heatmap was developed to represent taxonomic frequency and hierarchy by point at taxa level 3. The alpha diversity indices Chao1, Shannon, Simpson, Ace, and Pielou alpha diversity were calculated with the q2-diversity plugin to analyze species richness and evenness differences.

## 3. Results

### 3.1. Minimum Inhibition Concentration

Of the natural preservatives, ginger had the most minor antimicrobial activity, requiring >24 µL/100 µL concentration to inhibit the growth of tested strains (Table 1). Garlic had the lowest MIC (0.5–2 µL/100 µL) for the tested strains. Green tea showed 8 µL/100 µL for MIC. However, those three preservatives were rejected in the sensory evaluation. Ginger and green tea provided unacceptable color and appearance tests. Garlic was dismissed because of the intolerable odor test on the seven-point hedonic scale. Based on good MICs (Table 1) and acceptable sensory properties, the three natural preservatives we chose were malt vinegar, fresh lemon juice, and grapefruit seed extract (GSE) for the downstream study.

### 3.2. Bacterial Growth Curve for Store-Bought Fish

**Figure 1:**
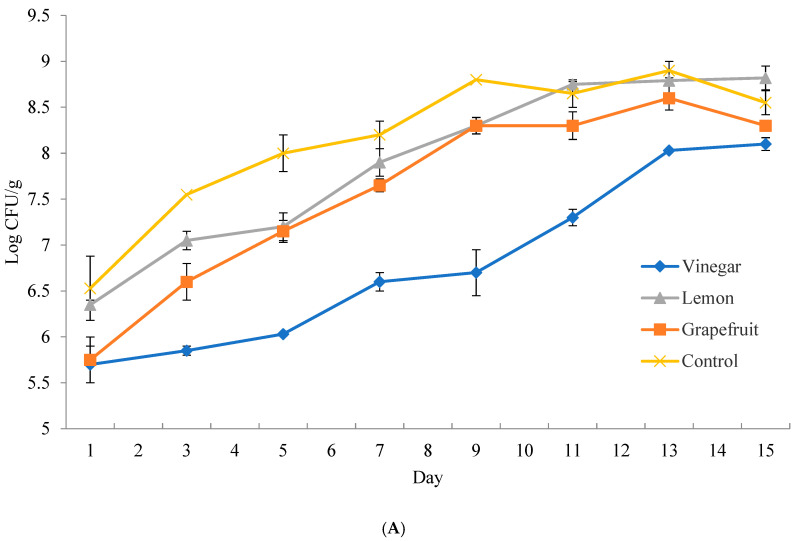

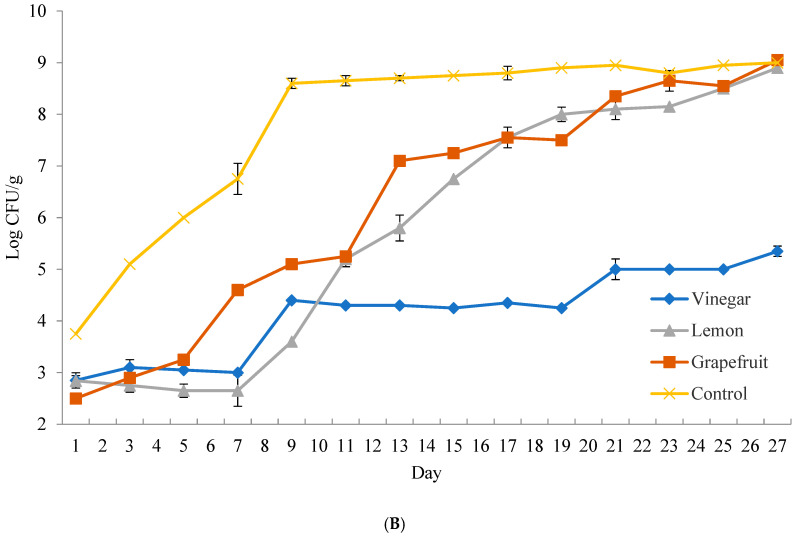
Bacterial growth of store-bought and aquaculture-raised fish samples. The total bacterial population of store-bought (**A**) and aquaculture-raised (**B**) fish and bacterial suppression due to natural preservative treatments was also observed. Samples were applied to TSA plates and were incubated at 26 °C.

On day one of store-bought catfish, the control total bacterial count was 6.5 Log CFU/g. After 30 min of marinating in each preservative, the total bacterial count was reduced to 6.3, 5.8, and 5.7 Log CFU/g for lemon, GSE, and vinegar treatments, respectively (Figure 1A). In subsequent days, an increasing gap developed between the treatment and control groups. By the endpoint (Day 15), the control and GSE-treated samples entered the death point, while lemon and vinegar-treated samples entered the stationary point.

Based on the bacterial growth, we noted “Early Point” for all samples on day one. We chose the “Middle Point’ on day seven for control and day nine for other treatments. “Late Point” on day fifteen was selected for all store-bought samples.

### 3.3. Bacterial Growth Curve for Aquaculture-Raised Fish

On day one, the control’s total bacterial count was 3.7 Log CFU/g in the aquaculture-raised catfish. After 30 min of marinating in each preservative, the total bacterial count was reduced to 2.8, 2.5, and 2.8 Log CFU/g for lemon, GSE, and vinegar treatments, respectively (Figure 1B). Unlike the store-bought catfish, the control entered the stationary point by day eleven and remained constant until the endpoint at day twenty-seven. As in the store-bought fish trial, lemon and GSE samples had similar bacterial growth patterns. At the midpoint of the control, all treatment samples showed effective antimicrobial activity by suppressing the bacterial growth to 2.3–4.5 Log CFU/g compared to the control growth of 6.7 Log CFU/g. However, unlike the other treatments, the bacterial population on the vinegar treatment samples grew very slowly throughout the trial. At the endpoint (Day 27), the vinegar treatment suppressed the growth of the bacterial population by almost 5 Log CFU/g compared to the control (9.1 Log CFU/g). The results showed that vinegar was the most effective antimicrobial treatment for store-bought and aquaculture catfish.

From these results, we chose “Early Point” for all samples starting on day one. We decided on the “Middle Point”, beginning on day seven for control aquaculture-raised samples and day eleven for other treatment aquaculture-raised samples. We chose “Late Point” starting on day eleven for control aquaculture-raised samples and day twenty-seven for other treatment aquaculture-raised samples.

### 3.4. Rarefaction Curves

From our NGS sequencing run, the Shannon index values for store-bought and aquaculture-raised samples reached a sufficient saturation at the sequencing depth of around 2500, respectively.

**Figure 2:**
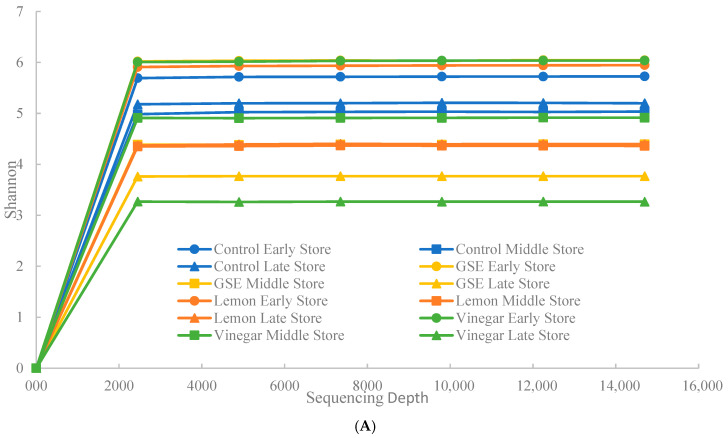

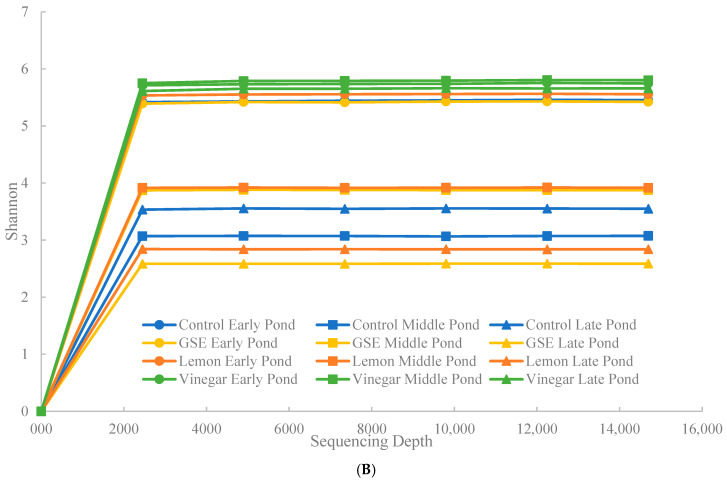
Rarefaction curves for store-bought (**A**) and aquaculture-raised (**B**) samples. Each sequencing depth was measured and compared using the Shannon index. Samples were treated with lemon, GSE, and vinegar at different storage points.

The rarefaction curves for the store-bought samples (Figure 2A) indicated higher Shannon index values for all early-point samples than other mid- and late points. The Shannon indices decreased in the vinegar late-point sample. The control middle and late point samples were lower than the control early point samples but were still higher than the treatment samples. Late-point treatment samples had lower Shannon index values than early and middle-point samples. From the aquaculture-raised samples, the values decreased as the storage day increased in the control and the treatments. In contrast, the rarefaction curves indicated high Shannon index values for all vinegar samples for the aquaculture-raised samples (Figure 2B). The late-point GSE samples had the lowest index values. Shannon index values decreased from the mid to late point for GSE and lemon treatment samples. However, the vinegar treatment samples in the middle and late points retained their high proportional taxonomy abundance, similar to the early point.

### 3.5. 16S rRNA Sequencing Data

A heat map (Supplementary Figure S2) was generated with the order level taxa table in QIIME 2, illustrating the frequency and hierarchical clustering of discovered bacteria among store-bought and aquaculture-raised samples. *Pseudomonadales* had a high frequency for most samples, followed by *Aeromonadales* and *Enterobacteriales*. There was a high frequency of *Flavobacteriales*, *Lactobacillales*, and *Burkholderiales* in early store-bought samples. Meanwhile, all the early aquaculture-raised samples, as well as middle- and late-point aquaculture-raised vinegar-treated samples, had a high frequency of *Bacteroidales*, *Flavobacteriales*, *Fusobacteriales*, and *Clostridilales*.

**Figure 3:**
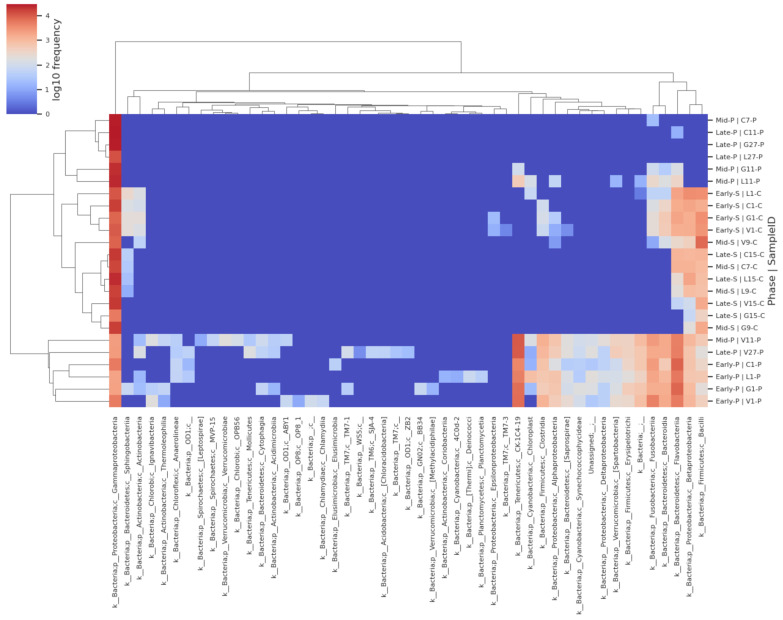
Heatmap of the relative abundance of each identified bacteria from the different treatments in the metagenomic sequencing analysis. The heatmap illustrates the visual display of taxonomic data from microbiome analysis. The *x*-axis shows the microbes at the class level. The *y*-axis shows the sample type, storage date, and natural preservative treatment. Hierarchy clustering was constructed to show the relative distance and complexity level in correspondent bacterial organisms (bottom) and sample type (right side).

We also generated a heat map (Figure 3) with the class-level taxa table in QIIME 2. *Gammaproteobacteria* (new phylum *Pseudomonadota)* had high frequency for most samples, followed by *Bacilli* and *Betaproteobacteria* (new phylum *Pseudomonadota*). There was a high frequency of *Flavobacteria* (new phylum *Flavobacteriota*), *Bacteroidia* (new phylum *Bacteroidota*), and *Fusobacteria* (new phylum *Fusobacteriota*) in early store-bought and aquaculture-raised samples and mid and late aquaculture-raised vinegar-treated samples. In addition to those classes, all the early aquaculture-raised samples and middle and late-point aquaculture-raised vinegar-treated samples had a high frequency of *CK-1C4-19* and *Clostridia*.

**Figure 4:**
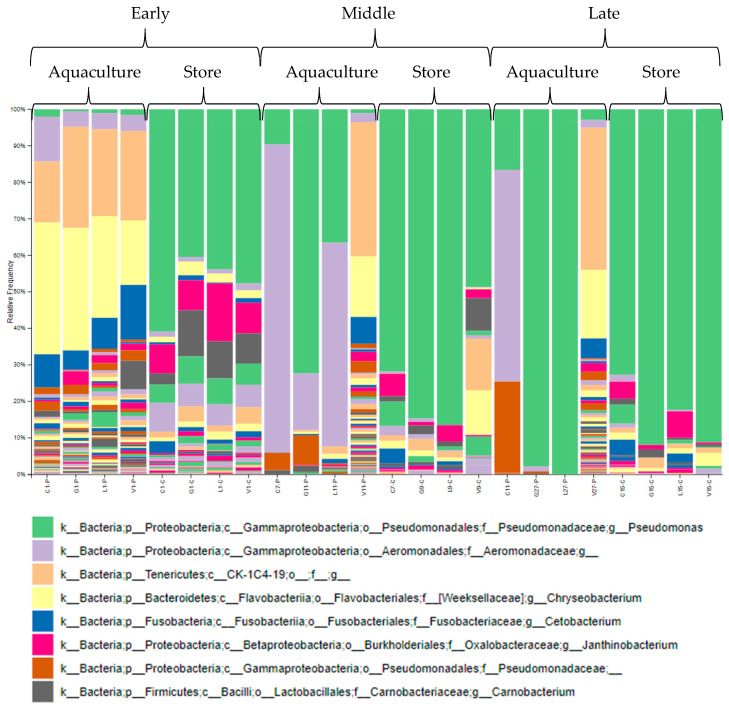
Microbial proportional abundance analysis. The bar plots show the relative abundance of microbiome from the kingdom to genus classification of store-bought (C) and aquaculture (P) fish samples for the early, middle, and late points for control (C) and preservative (G: GSE, L: lemon, and V: vinegar) treatments.

The bar plots for proportional taxonomy abundances were also generated from the phylum to the genus level for the store-bought samples (Figure 4). Among the communities, we chose the seven most abundant for further investigation. On day one, *Pseudomonas* accounted for 40–65% of microbe abundance in the store-bought samples and was higher in day one control samples versus treatment samples. Also, day one samples showed noticeable taxonomic abundances of *Janthinobacterium* and *Carnobacterium*. At the midpoint, *Pseudomonas* proportional abundances increased as the dominant genus in control, GSE-treated, and lemon-treated store-bought groups. The vinegar samples indicated no increase in *Pseudomonas* but showed an increase in other microbes, such as CK-1C4-19 and *Chryseobacterium*. By the endpoint, however, *Pseudomonas* dominated all store-bought treatment groups (>~72%).

For the aquaculture-raised samples, taxonomic classifications, and relative frequencies were generated up to the Genus level (Figure 4). Compared to the store-bought samples, on day one, *Pseudomonas* accounted for only 1–3% of microbes in the aquaculture samples, with most microbes being made up of a population of *CK-1C4-19*, *Chryseobacterium*, *Cetobacterium*, and the family *Aeromonadaceae*. The day-one aquaculture-raised sample had the least biased evenness among the major bacteria than the day-one store-bought sample. The population shifted over time, with *Aeromonadaceae* as the most abundant microbe except for vinegar samples in the middle point samples and ending with *Pseudomonas* as the most abundant microbe (>~97%) in the GSE and lemon samples. Proportional taxonomy abundance patterns for the aquaculture-raised vinegar-treated samples remained similar from early through late points, with most of the population being *Chryseobacterium*, *CK-1C4-19*, and *Cetobacterium*.

*Aeromonadaceae* were the most abundant microbes in the mid and late-points aquaculture-raised control samples, with *Pseudomonas* present to a lesser extent. A similar proportional taxonomy abundance sustained in the aquaculture-raised samples from the early and late points was maintained only in the vinegar treatment samples. Meanwhile, the aquaculture-raised control, GSE, and lemon samples became less diverse in the mid and late points.

Overall, *Aeromonadaceae* dominated the control aquaculture-raised samples over time, while *Pseudomonas* is prevalent in the control store-bought samples. The aquaculture-raised vinegar treatment samples sustained a similar evenness throughout the experiment, dominated by *CK-1C4-19*, *Chryseobacterium*, *Cetobacterium,* and *Janthinobacterium*. Interestingly, *Pseudomonas* and *Aeromonadaceae* never became dominant in the aquaculture-raised vinegar treatment samples and only reached <~4% in the total frequency. At the endpoint, the bacterial profile for the vinegar samples remained the same as at the early point. 

### 3.6. Alpha Diversity Indices

For the store-bought control samples (Table 2), most scores remained higher than the GSE, lemon, and vinegar treatments through storage duration. For the aquaculture-raised control samples, the Chao1 and ACE indices were high in the early points and declined during the middle and late points. As time passed, the aquaculture-raised GSE and lemon treatment samples had patterns comparable to those of the store-bought treatment samples. The aquaculture-raised vinegar treatment samples had high Chao1 and ACE index values that increased from early to late point, while Simpson index values remained steady throughout the experiment. Simpson index values slightly decreased in all store-bought samples and aquaculture-raised control, GSE, and lemon samples from early through late points.

In our study, most alpha diversity indices resulted within samples from early to late points were similar patterns except for the aquaculture-raised vinegar-treated samples. The number of other indices and Chao1 showed similar richness within the sampling points, suggesting that the sequencing depth was enough to catch all the alpha diversity in all samples. The aquaculture-raised vinegar-treated samples had consistent Shannon and Simpson indices and had the highest Chao1 and ACE index values of all samples. However, store-bought vinegar-treated samples had decreased alpha diversity and relative abundance in Shannon and Simpson index values from early through late points.

In Pielou, the greatest evenness was shown in the GSE early point from store-bought and lemon late point from aquaculture-raised samples. In contrast, lemon late and control middle points had the least Pielou evenness in store-bought and aquaculture-raised samples, respectively. The aquaculture-raised samples with vinegar treatment showed barely any changes of evenness during the entire storage period, while relative abundance in most other samples was significantly changed at the mid or late point.

### 3.7. Distance Matrix of Microbiome Between Catfish Samples

**Figure 5:**
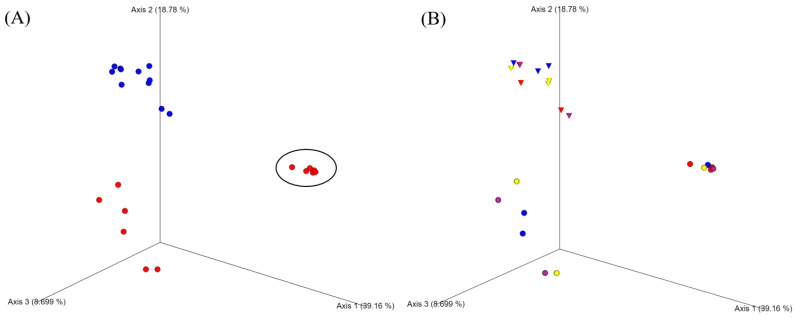
Principal coordinate (PCoA) analysis of control and natural preservative-treated samples. Unweighted Unifrac treatment plot for all fish samples shows distances based on similarities. (**A**) blue and red dots represent store-bought and aquaculture-raised samples, respectively. Circles encompass all vinegar-treated samples and early point control-, GSE-, and lemon-treated samples. (**B**) Cones and dots represent store-bought and aquaculture-raised samples. Each shape in blue, red, yellow, and purple refers to control, vinegar, lemon, and grapeseed treatment, respectively.

Beta diversity was observed between samples in the Principal Coordinate Analysis (PCoA) (Figure 5 and Supplementary Figure S3). The bacterial communities from each sample were plotted into three distinctive clusters: Unweighted/Weight Unifracs, Bray–Curtis, and Jaccard. From the Bray–Curtis based on sample type, store-bought and aquaculture-raised samples showed distinctive separation from each other and aquaculture-raised samples showed remarkable separation [23].

For the store-bought samples, control and all treatment samples for the early point were gathered in the unweight Unifrac and Jaccard distance, while samples for the mid and late points were separated from those samples. In the weight Unifrac, the store-bought control samples were grouped through storage duration, and the store-bought GSE and lemon samples in the middle and late points were exhibited at close distances with the late point sample for vinegar. 

From all the distance matrices based on similarity and storage point, aquaculture-raised vinegar-treated samples showed better relatedness than control, GSE, and lemon-treated samples. Overall, all the points of the aquaculture-raised vinegar samples showed a close distance from the early point of the aquaculture-raised control, lemon, and GSE samples. As the storage went to the middle and late points, the control, GSE, and lemon samples showed notable dissimilarities to those at the early point and all the vinegar samples.

## 4. Discussion

We chose fresh lemon juice, GSE, and vinegar for the minimum inhibition concentration based on their MIC and sensory response. Garlic was not selected because its efficacy varied from season to season, unlike the fresh lemon juice, which had consistent acidity from season to season. We did not include ginger and green tea because of sensory response concerns.

Organic monoprotic acids, such as vinegar, lemon, and grapefruit, donate one hydrogen ion per molecule when they dissociate in water, which may cause surrounding cells to pump in potassium ions, disrupting bacterial transmembrane proton motility, increasing intracellular osmotic pressure, and finally causing bacterial cytoplasmic membrane rupture [24]. Ayhan and Bilici showed that acetic acid disrupts the cell wall structure and thus causes a loss of ATP in the cell [25]. Another study found polyphenols disrupt the cell wall peptidoglycan structure and the phospholipid bilayer in the outer membrane of Gram-negative bacteria, impairing cell integrity, as well as interfering with the activity of intracellular bacterial enzymes by inhibiting the formation of amino and carboxyl groups of proteins [26]. These results, as well as our findings, support the hypothesis that polyphenols present in acetic acid possess antimicrobial activity against a broad spectrum of microorganisms [27,28].

Although lemon and GSE treatments exhibited antimicrobial activity, the vinegar treatments caused the greatest bacterial reduction of 2 Log CFU/g for store-bought fish and 5 Log CFU/g for the aquaculture-raised fish versus untreated samples. We suspect that the enhanced effectiveness of the vinegar (acetic acid) treatment on the aquaculture-raised catfish would be due to the initially lower bacterial load present on the aquaculture-raised catfish compared to the store-bought catfish.

The main bacteria on the aquaculture-raised catfish, including *Chryseobacterium*, *Aeromonadaceae,* and *Pseudomonas*, were susceptible to vinegar treatment. These bacteria are all Gram-negative and have a thin peptidoglycan cell wall [29]. It has been hypothesized that acetic acid is effective against Gram-negative bacteria because vinegar can cause a physical alteration of the less resistant cell wall caused by the thin murein layer [30]. It has also been suggested that the bactericidal effect of vinegar against Gram-negative bacteria may be related to the ability of the acids to pass through the bacterial membrane [31]. A study from another team showed a higher sensitivity of Gram-negative bacteria to vinegar in the growth phase than in the stationary phase [32]. This is similar to our results, in which vinegar decreased *Janthinobacterium* to low levels in store-bought samples from the early to the late point.

Previous research [1] reported that store-bought and aquaculture-raised catfish treated with malt vinegar resulted in minimal bacterial proliferation, remaining constant at approximately 6 Log CFU/g. In the present study, the vinegar treatment in the aquaculture-raised samples also effectively suppressed bacterial growth even at the endpoint.

All early point store-bought samples had less bias in proportional taxonomy abundance than middle and late-point samples. In the aquaculture-raised samples, all vinegar samples had less bias in proportional taxonomy abundance than others, followed by the remaining early, mid, and late point samples. Initially, some observed bacteria identified as spoilage bacteria, including *Pseudomonas* and *Aeromonadaceae*, increased during storage, and other bacterial proportional taxonomy abundances decreased. This is reflected in the store-bought and aquaculture-raised GSE and lemon treatment samples. The store-bought vinegar samples retained their proportional taxonomy abundance from the early to middle point. However, that proportional taxonomy abundance dropped off at the late point due to an increased biased relative abundance distribution by *Pseudomonas*. Meanwhile, high proportional taxonomy abundance and consistent evenness were present throughout storage duration in the aquaculture-raised vinegar samples.

In this study, the major bacteria observed were *Pseudomonas, Aeromonadaceae, Chryseobacterium, Cetobacterium, Janthinobacterium, Carnobacterium*, as well as *CK-1C4-19*. In the store-bought fish, GSE, lemon, and vinegar were able to reduce *Pseudomonas* during the early point only. Interestingly, *Janthinobacterium* and *Carnobacterium* growth, though also present in control and all treated samples in early point samples, became suppressed in middle and late point samples. In the aquaculture-raised control, GSE, and lemon-treated fish, the growth of *CK-1C4-19*, *Chryseobacterium*, *Cetobacterium*, and other bacteria decreased from the middle point. In contrast, the aquaculture-raised vinegar samples did not affect those bacteria. *Cetobacterium* is from the *Fusobacteriota* phylum, which are anaerobic bacteria and can ferment carbohydrates or amino acids and peptides, producing organic acids such as acetic acid [33], which may explain vinegar’s ineffectiveness against *Cetobacterium.* We hypothesize that the bacterial proportional taxonomy abundance and growth in this study are due to specific bacteria inhibited by components in the natural treatments, competitive suppression by consequent *Pseudomonas* growth, and simultaneous involvement of both factors. Further investigation is necessary.

From both store-bought and aquaculture-raised samples, most treatments, except for vinegar, were less effective in suppressing the growth of *Pseudomonas*, which became a dominant microorganism in the middle and late points. *Pseudomonas* are ubiquitous as they are found in various environments such as soil, water, plants, animal tissues, and foods [34]. *Pseudomonas* can also adapt to different conditions [35] due to their complex enzymatic systems [34,36,37]. Although they are aerobic, some can thrive with nitrate, fumarate, or other electron acceptors in oxygen-depleted environments [35]. Also, *Pseudomonas* can compete with other bacteria for iron due to their production of iron-binding siderophores. This is especially useful in iron-limited foods such as fish [35,38]. *Pseudomonas* spp. have been classified as SSOs in chilled meat [39] and other protein-rich food products such as dairy products [36,40], eggs [41], and freshwater fish [35,42]. *Pseudomonas* have become the dominant bacteria in refrigerated meats and have contributed significantly to spoilage due to their ability to break down glucose and amino acids [43]. According to Stanborough et al. [44], *P. fragi* contributes to the spoilage of fish, meat products, and ultra-pasteurized dairy products. *P. lundensis, P. fluorescens*, and *P. gessardii* also contribute to the spoilage of milk products [35].

This study found the lactic acid bacteria (LAB) *Carnobacterium* in all early and mid-point store-bought samples. LAB can dominate the microflora of chilled fish products in vacuum packs when treated with salt (NaCl) and acids [38]. *Lactobacillus* can grow in seafood treated with acids and other chemical preservatives [38]. *Leuconostoc* also contributes to refrigerated raw meat spoilage [43]. Under specific conditions, LAB are also competitors of other spoilage-related microbial groups [43].

In the aquaculture-raised samples, we identified other notable spoilage bacteria, such as those from class *CK-1C4-19* and phylum *Fusobacteriota*. The bacteria *CK-1C4-19* has been found in blue mussels [45], crabs [46,47], salmon [48], sea stars [49], minnows [50], and zebrafish [51]. *Fusobacteria* has been found in rainbow trout [52], carp [53], catfish, bass, and bluegill [54,55]. Both have been shown to contribute to oyster spoilage [56,57].

Hickey et al. [58] suggested in a previous study that the rapid deterioration of store-bought fish compared to aquaculture-raised fish at refrigerated storage might be due to mishandling tissue during farm-to-table processing. The different sources of seafood products [59,60] and transport time to markets [61,62] can also influence the taxonomy of bacterial composition detected from the samples and the change due to the application of natural preservatives.

For the alpha diversity indexes, lower values were assigned to samples in a less diverse system where only a few genera dominate the population [63]. This study also observed in store-bought treated catfish samples where *Pseudomonas* was dominant towards the middle and late points. The control samples in store-bought and aquaculture-raised fish initially had high richness. From the middle point, the aquaculture-raised control sample demonstrated decreased richness and increased evenness of the most abundant bacteria. In contrast, the store-bought control samples showed fewer changes in richness and evenness than those of the aquaculture-raised control sample. Interestingly, the aquaculture-raised vinegar treatment samples resulted in minor changes in Simpson, Shannon, and Pielou values in contrast to slight increases in chao 1 and ACE values after day one, which might be more sensitive to rare OTUs [64]. This complements the taxonomic data where many of the same genera identified on day one were seen on the final day in a different proportional distribution and agreed with previous data from other labs, demonstrating a negative correlation between evenness and richness [65,66,67,68].

The heatmap (Figure 3) shows the difference in bacteria abundance between aquaculture-raised and store-bought catfish. Previous studies [69,70,71] have shown differences in bacteria from fish from different sources, likely due to differences in diet and environment.

The heat map with the class-level taxa exhibited that aquaculture-raised and store-bought catfish had considerable amounts of *Gammaproteobacteria, Bacilli*, and *Betaproteobacteria*. The frequency of *Pseudomonadaceae,* a family member of class *Gammaproteobacteria,* was higher in all store-bought and middle and late-point aquaculture-raised samples than in others. *Pseudomonadaceae* has previously been found in fish and is common in fresh and marine waters [72]. *Aeromonadaceae* are closely related to *Enterobacteriaceae* and are both other family members under class *Gammaproteobacteria*. They have shared biochemical features and ecosystems [73].

In the order level, early aquaculture-raised fish additionally had a high frequency of *CK-1C4-19*, *Flavobacteriales* and *Fusobacteriales*, and *Bacteroidales*. Still, the frequency of these bacteria decreased through the middle and late points except for the vinegar-treated samples. *Fusobacteriales* and *Bacteroides* have previously been found in other types of fish [71]. In the family-level taxa, store-bought fish frequently had *Carnobacteriaceae*, *Listeriaceae*, *Oxalobacteraceae, Flavobacteriaceae,* and *Streptococcaceae* throughout storage duration (refer to the Supplementary Figure S2B). *Streptococcaceae* has been found previously in other types of fish [74,75].

Unweighted and Weighted Unifrac, Bray–Curtis, and Jaccard dissimilarity were used to study the distance of bacterial communities between samples in distinct points. The PCoA charts demonstrated good clustering of store-bought samples but two different aquaculture-raised sample clusters (Figure 5). This may be due to the homogeneous communities of the store-bought samples, because of the strict regulations around fish product husbandry, fillet processing, and the potential for biased bacterial growth during transportation and market sales. Overall, there was good clustering of each type of sample treatment, demonstrating each preservative’s effectiveness against specific bacteria. Primarily, there were close community similarities between the aquaculture-raised vinegar samples and the fresh early point control sample in Unifrac, Bray–Curtis, and Jaccard PCoAs (Figure 5). These coordinates demonstrate reflected similarity and change tracking among bacterial communities in β-diversity [76].

We used Next-Generation Sequencing (NGS) metagenomics to analyze the microbiome of store-bought and aquaculture-raised catfish fillets treated with natural preservatives (vinegar, lemon, and grapefruit seed extract) during refrigerated storage. The bacterial communities were sequenced using the Illumina system, and data were analyzed with the QIIME2 pipeline. We found that vinegar was the most effective preservative, significantly reducing bacterial growth and extending the shelf-life of catfish.

This research provides technological insights into the use of NGS for food microbiology, highlighting its ability to offer specific details about the microbiome and their dynamics. Vinegar is a cost-effective preservative that can be scaled for larger operations, offering a natural alternative to chemical preservatives. Vinegar is also a natural preservative, which aligns with consumer preferences for less processed foods with fewer chemical additives. Vinegar may affect certain nutrients, but the tradeoff for reduced spoilage bacterial growth is beneficial for food safety. This work underscores the potential of natural preservatives to improve food safety, quality, and shelf-life, contributing to the ability of the fishing industry to deliver high-quality fish products to consumers.

## 5. Conclusions

In conclusion, of the three treatments, vinegar caused the most significant bacteria reduction at the midpoint growth (99%) in the store-bought fish and the aquaculture-raised fish from the mid to the late point (>99%). *Pseudomonas* dominated the other bacteria at the early point for all sample treatments in the store-bought fish. However, *Pseudomonas* was suppressed by the vinegar treatment until the middle point, allowing for more evenness from proportional taxonomy abundance. In the aquaculture-raised fish, *Pseudomonas* dominated other bacteria in the sample treatments for the mid and late points but never became the dominant genera for the aquaculture-raised vinegar samples. Except for vinegar and fresh early-point samples, all early-point samples in the store-bought and aquaculture-raised fish exhibited less microbial evenness. However, the vinegar samples maintained better proportional taxonomy abundance scores in the aquaculture-raised fish samples. All early-point aquaculture samples showed elevated levels of *CK-1C4-19*, *Chryseobacterium*, and *Cetobacterium*. Both GSE and lemon effectively decreased the numbers of these bacteria while allowing *Pseudomonas* to become the dominant genus in the middle and late points. Likewise, while *Janthinobacterium*, *Carnobacterium*, and *Aeromonadaceae* were present in the early point store samples, through the middle and late point samples, GSE and lemon treatment decreased those bacteria but were not effective in controlling *Pseudomonas*. It has been shown that *Pseudomonas* becomes the dominant species during fish processing [77,78], possibly because the bacteria find more favorable conditions in the matrix than in the outside environment [79].

Our study found vinegar to be most effective in increasing shelf-life, while GSE and lemon were less effective in storing catfish fillets. Although vinegar has been shown to have some negative effects on certain essential nutrients in fruits and vegetables [80], we believe the tradeoff for decreased bacterial growth is worthwhile in fish products. This effect was seen even without negative changes in sensory evaluation. Additionally, since vinegar is more cost-effective than other natural and synthetic preservatives, it offers advantages over commonly used counterparts and can be scaled to larger operations. We also found that using a metagenomics approach to study preservatives’ effectiveness against SSOs and foodborne bacteria can provide robust information on the effects of natural preservatives on specific bacteria along with diversities vs. conventional food preservative studies that study only a limited bacteria species growth or only growth of a general bacteria population [81]. This knowledge would provide an understanding of the dynamics of microbiota on fish spoilage and could suggest effective natural preservative treatments to extend the shelf-life of fishery products. As ongoing studies, future investigations will focus on food spoilage associated with SSOs’ adaptation and behavior, using omics approaches.

## Figures and Tables

**Table 1:** Minimum inhibition concentration (µL/100 µL) of natural preservatives to evaluate SSOs.

Bacteria	Lemon (6.7% Acid)	Grapefruit	Vinegar (5% Acid)	Green Tea	Ginger	Garlic
*Shewanella algae* ATCC51192	4 (0.27% acid)	8 (2.64% grapefruit)	4 (0.2% acid)	4	>24	0.5
*Shewanella baltica* NCTC10735	4	8	2	4	>24	0.5
*Aeromonas hydrophilia* ATCC 7966	8	>8	4	8	>24	2
*Aeromonas salmonicida* ATCC 33658	4	8	2	2	>24	1
*Pseudomonas aeruginosa* ATCC 10145	>8	>8	4	>8	>24	2
*Pseudomonas fluorescens* ATCC 13525	8	8	4	4	>24	2

**Table 2:** Alpha diversity indices for aquaculture-raised and store-bought catfish samples. The first letter corresponds to treatment (C: control, G: grapefruit, V: vinegar, L: lemon), the number corresponds to sampling days, and the last letter corresponds to the source (C: store-bought, P: aquaculture-raised).

	Point	chao1	ace	Simpson	Shannon	Pielou
C1-C	Early	140.00	140.00	0.97	5.73	0.80
C7-C	Mid	90.00	90.35	0.95	5.04	0.78
C15-C	Late	99.00	99.00	0.95	5.21	0.79
G1-C	Early	129.00	129.29	0.98	6.05	0.86
G9-C	Mid	63.00	63.00	0.93	4.40	0.74
G15-C	Late	23.00	23.00	0.91	3.77	0.83
V1-C	Early	135.50	135.46	0.98	6.04	0.85
V9-C	Mid	65.00	65.00	0.96	4.92	0.82
V15-C	Late	25.00	25.00	0.86	3.27	0.70
L1-C	Early	127.00	127.35	0.98	5.95	0.85
L9-C	Mid	63.00	63.00	0.92	4.37	0.73
L15-C	Late	88.00	88.00	0.92	4.40	0.68
C1-P	Early	129.00	129.00	0.96	5.46	0.78
C7-P	Mid	41.00	42.48	0.83	3.07	0.59
C11-P	Late	37.00	40.00	0.89	3.55	0.69
G1-P	Early	134.50	135.06	0.96	5.43	0.77
G11-P	Mid	36.00	36.00	0.91	3.88	0.75
G27-P	Late	17.00	17.00	0.81	2.59	0.63
V1-P	Early	151.00	151.00	0.97	5.75	0.79
V11-P	Mid	187.00	187.00	0.96	5.81	0.77
V27-P	Late	167.00	167.00	0.96	5.66	0.77
L1-P	Early	115.00	115.00	0.97	5.56	0.81
L11-P	Mid	43.00	43.00	0.91	3.92	0.72
L27-P	Late	8.00	8.00	0.86	2.84	0.95

## Data Availability

The original contributions presented in this study are included in the article/Supplementary Material. Further inquiries can be directed to the corresponding author.

## Supplementary Materials

The following supporting information can be downloaded at: https://www.mdpi.com/article/10.3390/microorganisms13020244/s1, Figure S1: Experimental design of the experiment. Figure S2: (A,B) Heatmap of the relative abundance of each identified bacteria from the different treatments in the metagenomic sequencing analysis. Figure S3: Principal coordinate (PCoA) analysis of control and natural preservative-treated samples using Weighted Unifrac, Bray–Curtis, and Jaccard metrics. Figure S4: Sensory attributes rating average of catfish fillets. Table S1: Taxonomic composition and relative abundance.
