# Supplementary material for: The Microbiome of Catfish (Ictalurus punctatus) Treated with Natural Preservatives During Refrigerated Storage
Source: Microorganisms. 2025 Jan 23;13(2):244. doi: 10.3390/microorganisms13020244 (PMC11857556; doi:10.3390/microorganisms13020244)
Supplement: Supplementary file 1 [file microorganisms-13-00244-s001.zip › microorganisms-3417415-supplementary.pdf]

Supplementary Materials

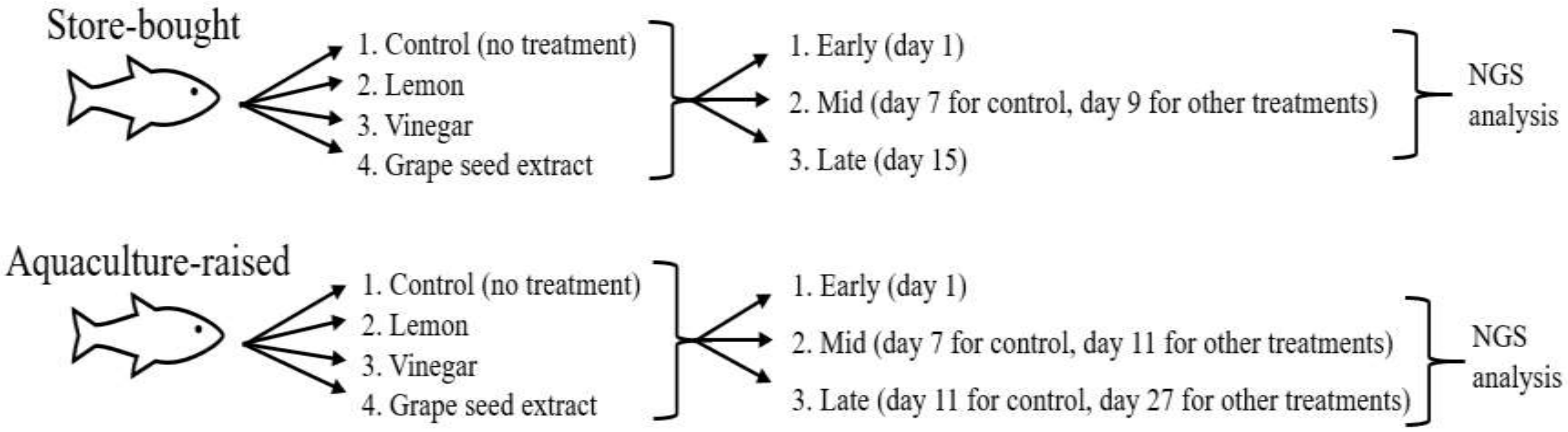

Figure S1. Experimental design of the experiment. Store-bought and aquaculture-raised catfish were treated with natural preservatives under four conditions: 1. control, 2. lemon, 3. vinegar, and 4. grape seed extract. The fish were then stored for 1 to 27 days, representing early, mid, and late storage points. After storage, the samples were analyzed using Next-Generation Sequencing.

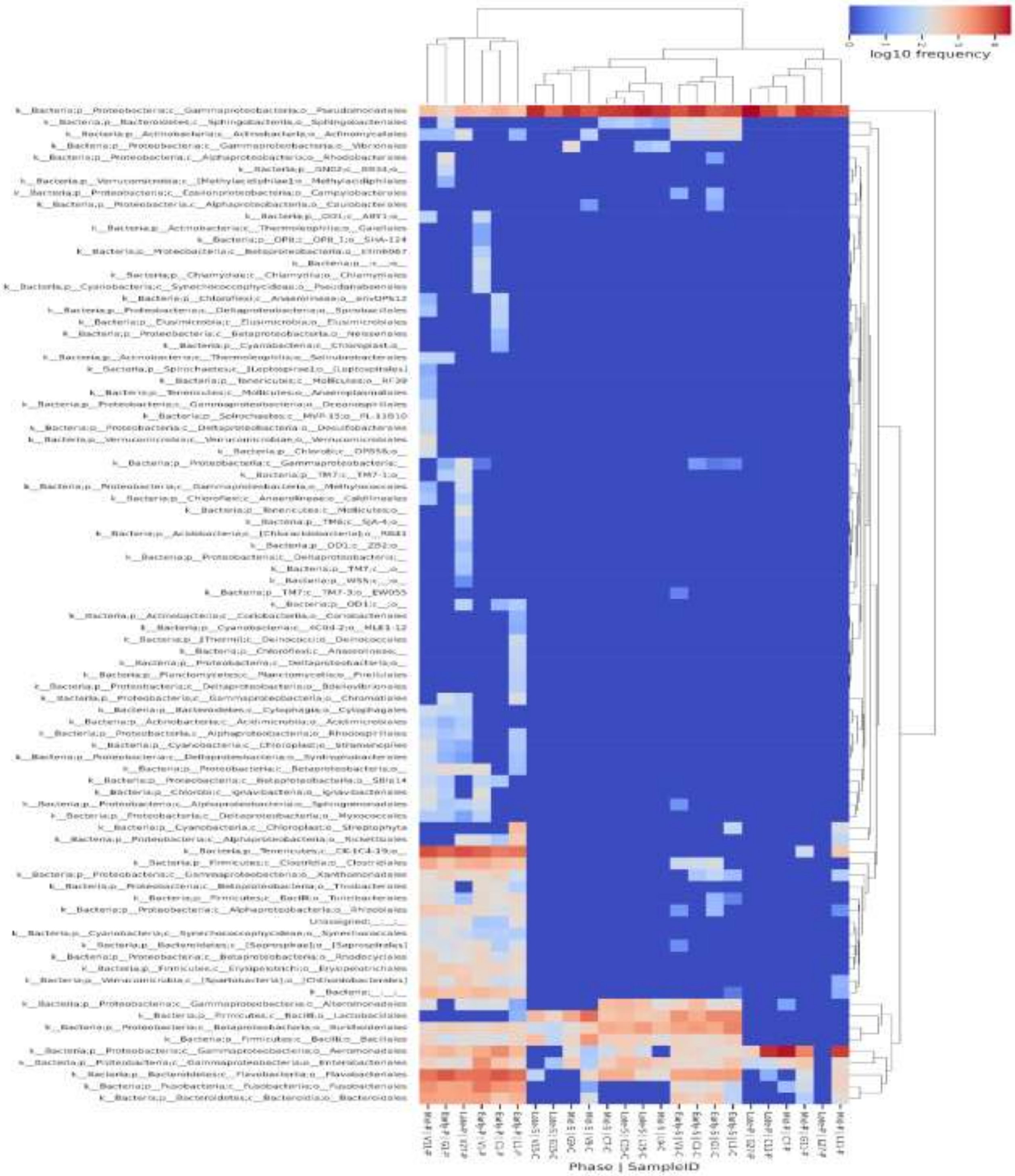

(**A**)

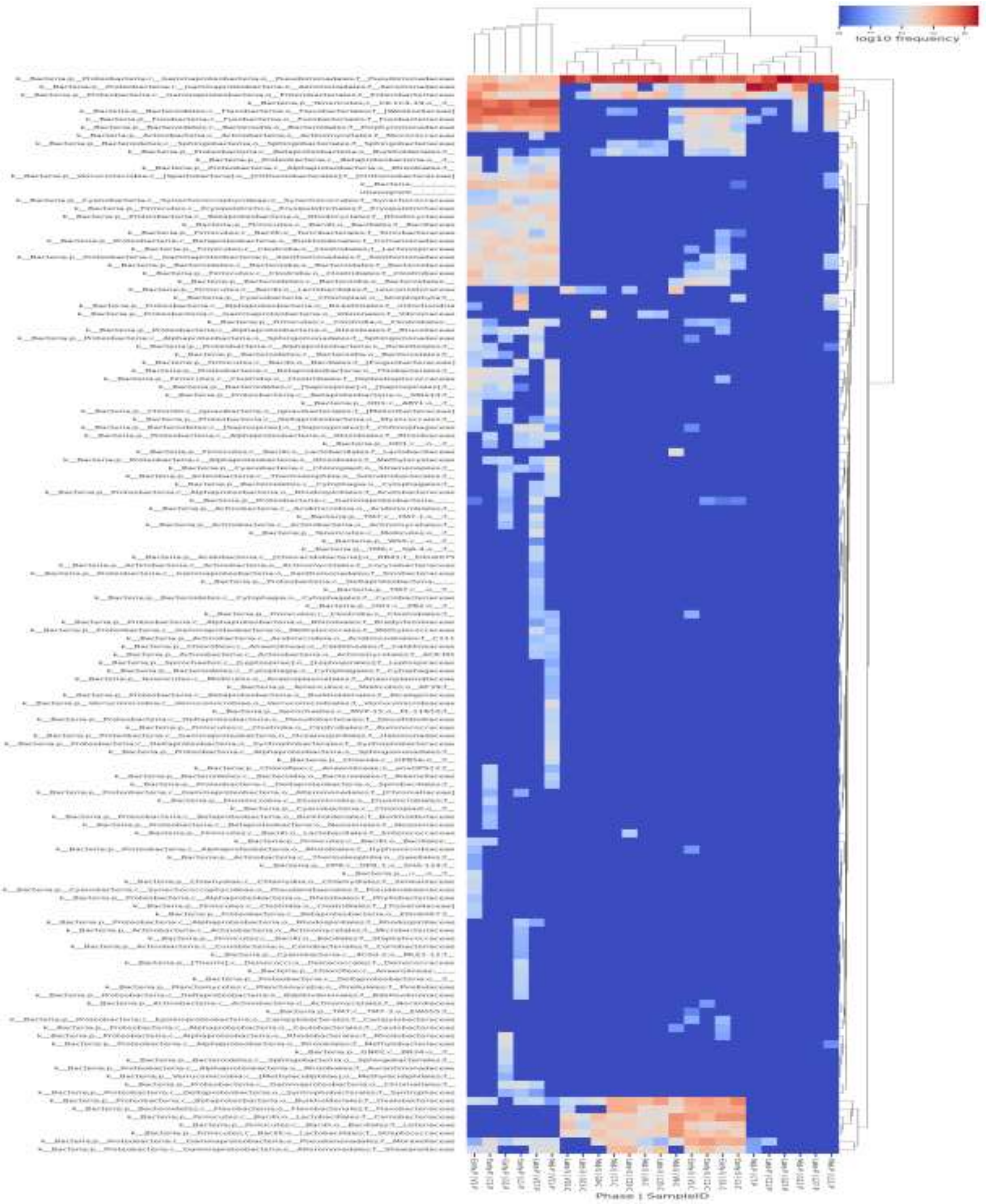

(B)

Figure S2 (A). Heatmap of the relative abundance of each identified bacteria from the different treatments in the metagenomic sequencing analysis. The heatmap illustrates the visual display of taxonomic data from microbiome analysis. The y-axis shows the microbial species at the order level. The x-axis shows the sample type, storage date, and natural preservative treatment.

Hierarchy clustering was constructed to show the relative distance and complexity level in correspondent bacterial organisms and sample type. (B). Heatmap of the relative abundance of each identified bacteria from the different treatments in the metagenomic sequencing analysis. The heatmap illustrates the visual display of taxonomic data from microbiome analysis. The y-axis shows the microbial species at the family level. The x-axis shows the sample type, storage date, and natural preservative treatment. Hierarchy clustering was constructed to show the relative distance and complexity level in correspondent bacterial organisms and sample type.

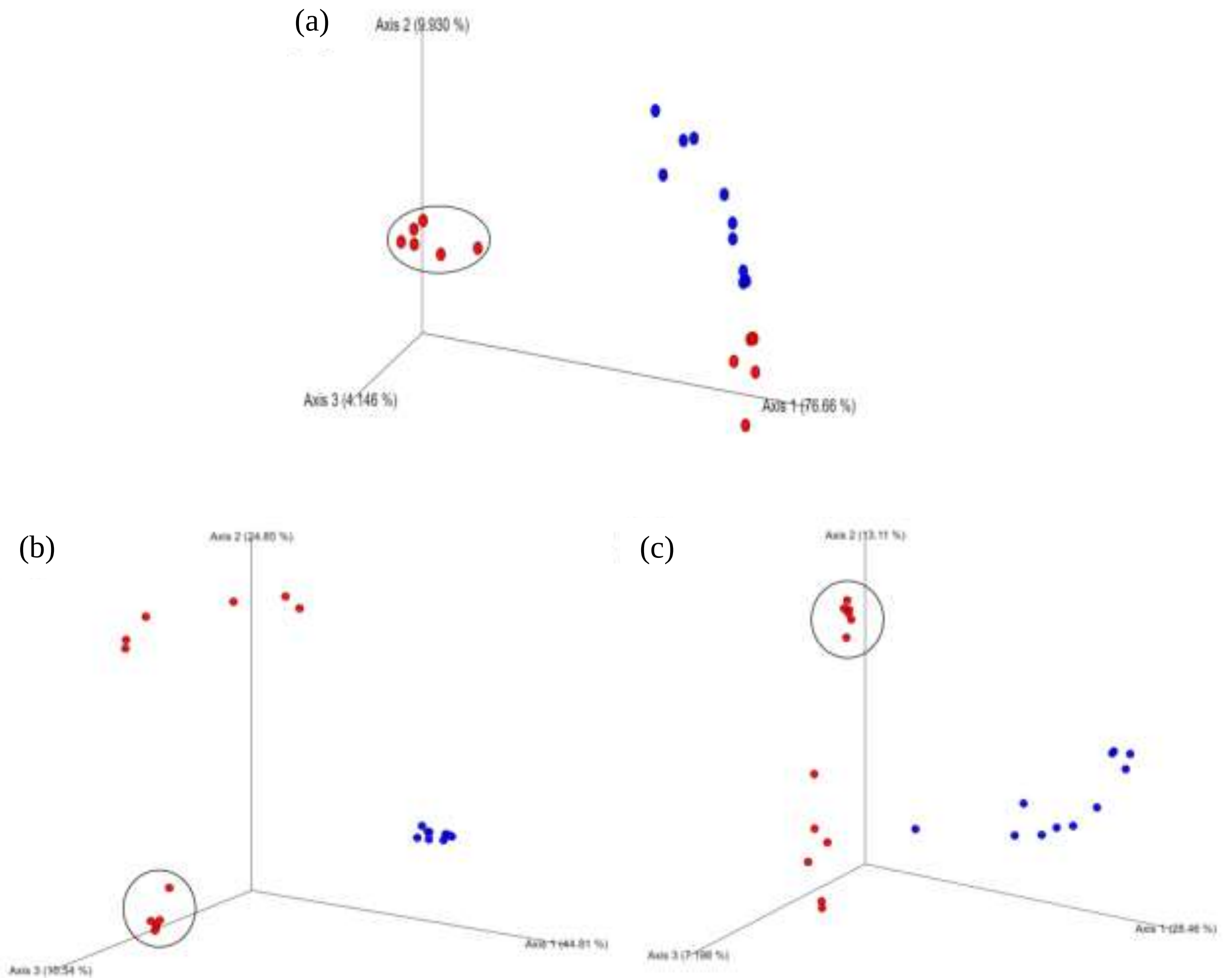

Figure S3. Principal coordinate (PCoA) analysis of control and natural preservative-treated samples. The Weighted Unifrac (a), Bray-Curtis (b), and Jaccard (c) treatment plots for all fish samples show distances based on similarities. Blue and red dots represent store-bought and aquaculture-raised samples, respectively. Circles encompass all vinegar-treated samples and early point control-, GSE-, and lemon-treated samples.

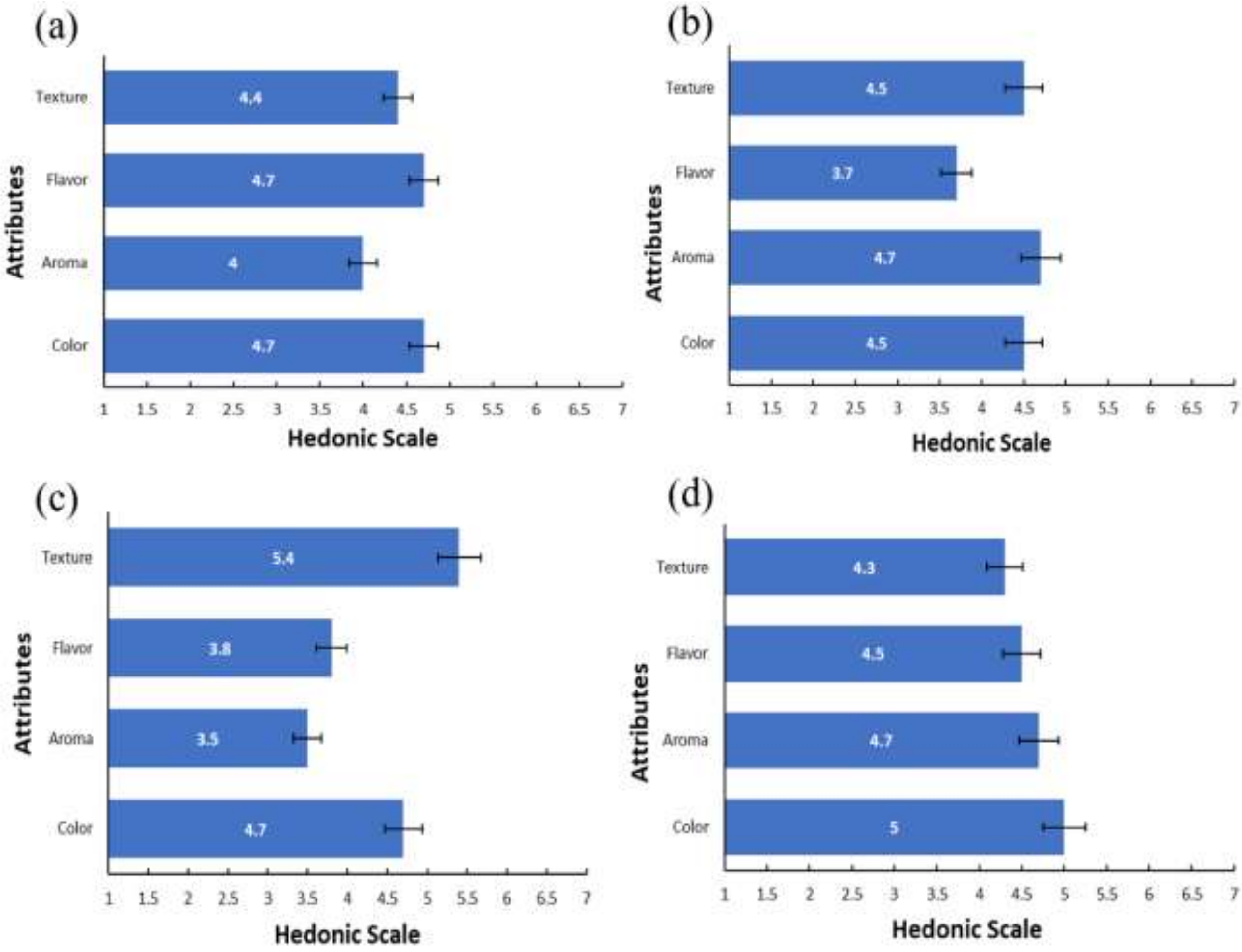

Figure S4. Sensory attributes rating average of catfish fillets. Sensory evaluations, focusing on affective testing for appearance (color), aroma, flavor, and texture, were conducted using a 7-point hedonic scale. Control (a), GSE (b), lemon (c), and vinegar (d) treatments.

Table S1. Taxonomic composition and relative abundance from the kingdom to genus.

SampleType	Treatment	Day	Phase	index	[illegible] bacteria;c__Gammaproteobacteria;o__Pseudomonadales;f__Pseudomonadaceae;g__Pseudomonas	k__Bacteria;p__Proteobacteria;c__Gammaproteobacteria;o__Aeromonadales;f__Aeromonadaceae;g__	k__Bacteria;p__Tenericutes;c__CK-1C4-19;o__;f__;g__	k__Bacteria;p__Bacteroidetes;c__Flavobacteriia;o__Flavobacteriales;f__[Weeksellaceae];g__Chryseobacterium	k__Bacteria;p__Fusobacteria;c__Fusobacteriia;o__Fusobacteriales;f__Fusobacteriaceae;g__Cetobacterium	k__Bacteria;p__Proteobacteria;c__Gammaproteobacteria;o__Pseudomonadales;f__Pseudomonadaceae;__	[illegible] bacteria;c__Betaproteobacteria;o__Burkholderiales;f__Oxalobacteraceae;g__Janthinobacterium	k__Bacteria;p__Firmicutes;c__Bacilli;o__Lactobacillales;f__Carnobacteriaceae;g__Carnobacterium
Aquaculture	Control	1	Early-P	C1-P	2.01%	12.41%	16.68%	36.09%	9.10%	1.87%	0.00%	0.00%
Aquaculture	GrapeSeed	1	Early-P	G1-P	0.50%	4.36%	27.68%	33.59%	5.32%	0.21%	0.00%	0.00%
Aquaculture	Lemon	1	Early-P	L1-P	1.02%	4.55%	23.78%	27.85%	8.50%	0.92%	0.00%	0.00%
Aquaculture	Vinegar	1	Early-P	V1-P	1.52%	4.48%	24.52%	17.68%	14.98%	0.65%	0.00%	0.00%
StoreBought	Control	1	Early-S	C1-C	60.99%	1.39%	0.00%	1.52%	0.61%	0.00%	7.90%	3.09%
StoreBought	GrapeSeed	1	Early-S	G1-C	40.55%	1.27%	0.00%	3.74%	1.37%	0.00%	8.19%	12.66%
StoreBought	Lemon	1	Early-S	L1-C	43.96%	1.12%	0.00%	2.46%	0.25%	0.00%	15.80%	10.19%
StoreBought	Vinegar	1	Early-S	V1-C	47.80%	1.87%	0.00%	2.14%	1.27%	0.00%	8.36%	8.27%
Aquaculture	Control	7	Mid-P	C7-P	9.71%	84.38%	0.00%	0.00%	0.07%	4.70%	0.00%	0.00%
Aquaculture	GrapeSeed	11	Mid-P	G11-P	72.45%	15.53%	0.42%	0.62%	0.39%	8.09%	0.00%	0.00%
Aquaculture	Lemon	11	Mid-P	L11-P	36.64%	55.80%	2.06%	1.33%	1.19%	0.41%	0.00%	0.00%
Aquaculture	Vinegar	11	Mid-P	V11-P	1.05%	2.45%	36.90%	16.57%	7.37%	1.18%	0.00%	0.00%
StoreBought	Control	7	Mid-S	C7-C	71.93%	0.50%	0.00%	0.06%	0.00%	0.00%	6.14%	1.52%
StoreBought	GrapeSeed	9	Mid-S	G9-C	84.77%	0.93%	0.00%	0.00%	0.00%	0.00%	0.94%	2.49%
StoreBought	Lemon	9	Mid-S	L9-C	86.62%	0.00%	0.00%	0.00%	0.00%	0.00%	4.46%	1.33%
StoreBought	Vinegar	9	Mid-S	V9-C	48.78%	0.19%	0.00%	0.40%	0.05%	0.02%	2.38%	8.94%
Aquaculture	Control	11	Late-P	C11-P	16.68%	57.93%	0.00%	0.04%	0.00%	24.96%	0.00%	0.00%
Aquaculture	GrapeSeed	27	Late-P	G27-P	97.86%	1.35%	0.00%	0.00%	0.00%	0.53%	0.00%	0.00%
Aquaculture	Lemon	27	Late-P	L27-P	100.00%	0.00%	0.00%	0.00%	0.00%	0.00%	0.00%	0.00%
Aquaculture	Vinegar	27	Late-P	V27-P	2.92%	2.21%	38.94%	18.78%	5.40%	0.31%	0.00%	0.00%
StoreBought	Control	15	Late-S	C15-C	72.84%	1.79%	0.00%	0.04%	0.00%	0.00%	4.69%	1.64%
StoreBought	GrapeSeed	15	Late-S	G15-C	92.03%	0.00%	0.00%	0.00%	0.00%	0.00%	1.18%	2.28%
StoreBought	Lemon	15	Late-S	L15-C	82.44%	0.37%	0.00%	0.00%	0.00%	0.01%	7.22%	0.59%
StoreBought	Vinegar	15	Late-S	V15-C	91.32%	0.00%	0.00%	0.00%	0.00%	0.00%	0.45%	0.76%
